# Photoluminescence characteristics of Cd_1-*x*_Mn*_x_*Te single crystals grown by the vertical Bridgman method

**DOI:** 10.1186/1556-276X-7-36

**Published:** 2012-01-05

**Authors:** Younghun Hwang, Youngho Um, Hyoyeol Park

**Affiliations:** 1 Basic Science Research Institute, University of Ulsan, Ulsan, 680-749, South Korea; 2 Department of Physics, University of Ulsan, Ulsan, 680-749, South Korea; 3 Department of Semiconductor Applications, Ulsan College, Ulsan, 680-749, South Korea

**Keywords:** Cd_1-*x*_Mn*_x_*Te, Bridgman method, PL, exciton, broadening, thermal quenching

## Abstract

In this paper, we report a systematic investigation of band-edge photoluminescence for Cd_1-*x*_Mn*_x_*Te crystals grown by the vertical Bridgman method. The near-band-edge emissions of neutral acceptor-bound excitons (labeled as L1) were systematically investigated as a function of temperature and of alloy composition. The parameters that describe the temperature variation of the energy were evaluated by the semiempirical Varshni relation. From the temperature dependence of the full width at half maximum of the L1 emission line, the broadening factors *Γ*(*T*) were determined from the fit to the data. The activation energies of thermal quenching were obtained for the L1 peak from the temperature dependence of the bound exciton peaks and were found to decrease with increasing Mn concentration.

## Introduction

Diluted magnetic semiconductors [DMSs] are semiconductor alloys formed by randomly replacing a fraction of the cations in a compound semiconductor with magnetic ions, e.g., Mn^2+ ^in CdTe to form Cd_1-*x*_Mn*_x_*Te [1]. The presence of the magnetic ions leads to a number of unusual electronic, optical, and magneto-optical properties, including the ability to magnetically tune the band gap made possible in this material by the large *sp-d *exchange interaction between magnetic ions and band electrons [2]. These properties make Cd_1-*x*_Mn*_x_*Te [CMT] promising candidates for fabricating magneto-optical devices, such as Faraday rotators, isolators, magneto-optical switches, and solar cells [3]. Recently, CMT has attracted much interest as a potential material for applications in the gamma-ray detectors because of a wide band gap and high resistivity [4]. The bulk CMT crystals have been grown with different growth methods, such as vertical gradient freeze, traveling heater, and vertical Bridgman method. Among these, the vertical Bridgman technique can be widely used successfully to grow CMT bulk crystals. The CMT crystals fabricated by this method showed zinc-blende structures for *x *< 0.77 [1]. Photoluminescence [PL] is a useful technique that has been employed to characterize bulk materials [5]. This technique has been used to study the near-band-edge excitonic states and structural quality in materials. In particular, the temperature dependence of PL spectrum intensity has been used to obtain information about electronic gap levels in semiconductors.

In this work, we present the PL study on the CMT bulk crystals grown by the vertical Bridgman method to investigate the variation of the band-edge transition energy in the PL spectra as a function of Mn composition *x *and temperature. In addition, the measurements made it possible to analyze and discuss in detail the broadening mechanisms of the exciton emission lines and their dependence on both the Mn content and temperature.

### Experiment

CMT crystals were grown by using the vertical Bridgman method from Cd(6N), Te(6N), and Mn(4N) elements. The elements were vacuum-sealed in a carbon-coated quartz ampule under a pressure of 1 × 10^-6 ^Torr. The reaction temperature was slowly raised from 600°C to 1,200°C. The ampule was held at 1,200°C for 3 h to homogenize the melt and lowered at a rate of 1.44 mm/h. The solidification gradient in the furnace was 22.5°C/cm. The crystal obtained was of cylindrical form of 10 mm in diameter and 20 mm long. The mole fraction *x *was determined by an electron probe microanalyzer (EPMA-1400; Shimadzu Corporation, Nakagyo-ku, Kyoto, Japan). Before PL measurements, CMT samples were cut into 5 × 5 × 1 mm^3^, mechanically polished, and then etched in 2% bromine-methanol solution. In order to measure the PL spectra, the samples were cooled down to 12 K in a cryogenic system and excited by a 442-nm line of a He-Cd laser (Liconix 4240NB; Sunnyvale, CA, USA). The luminescence was detected by a photomultiplier tube using a grating monochromator and analyzed by a computer-aided data acquisition system.

## Results and discussion

**Figure 1:**
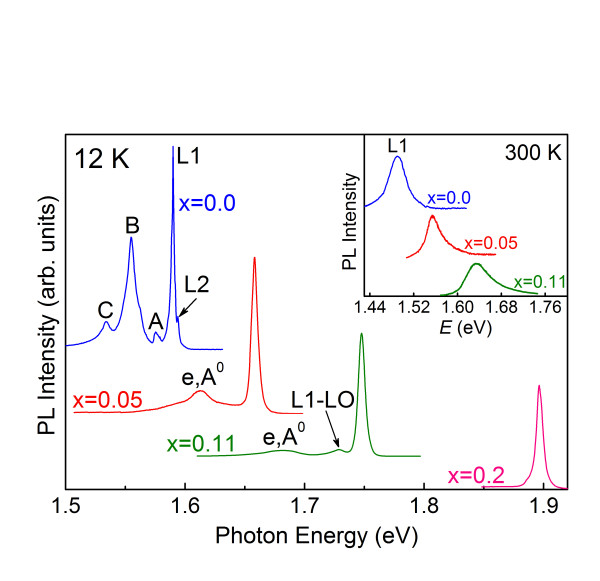
**Photoluminescence spectra observed at 12 K**. The spectra was observed for a series of CMT crystals with different Mn concentrations *x*. The inset displays typical PL spectra at 300 K.

Figure 1 shows the PL spectra at 12 K and 300 K taken on the CMT crystals for Mn concentration from *x *= 0.0 to 0.2. In DMSs, the strong *sp-d *exchange interaction between carriers and localized magnetic ions gives rise to the formation of a magnetic polaron and causes an energy shift as a result. However, we only considered the behavior of the exciton peak with temperature and composition without taking the formation of a magnetic polaron into account because our temperature was slightly high to show the bound magnetic polaron in the PL spectra, and there was no applied magnetic field. The PL spectra are dominated by exciton peaks, i.e., there is no noticeable luminescence associated with self-activated or other deep centers. The band-edge emission peaks of a CdTe crystal are similar to those found in the PL spectra reported by other investigators [6,7], and it will therefore be convenient to use our results for CdTe as a point of departure.

The inset of Figure 1 shows the PL spectra for CMT with different Mn mole fractions at 300 K. The line shapes of the spectra taken at 300 K consist of a single band which corresponds to transitions across the band gap. The intense PL emission at 300 K in CdTe reflects that the samples are high quality crystals. The dominant emission peak observed for CdTe at 1.590 eV is attributed to neutral acceptor-bound exciton transitions (labeled L1). This acceptor bound exciton feature dominates in the PL spectrum observed from a range of grown CdTe samples which are usually *p*-type [8]. We ascribe the weaker higher-energy feature at 1.594 eV associated with bound exciton recombination about shallow donors (labeled L2) [9]. The spectra also show a weak luminescence peak at about 1.575 eV, which is identified as the first longitudinal-optical [LO]-phonon replica (designated as A) of the free exciton transition [7]. The spectra also show weak luminescence peaks at about 1.555 and 1.534 eV, which we interpreted to be the second and third LO-phonon replicas (designated as B and C) of the free exciton [7]. The general features of the CdTe PL spectra are also seen for the CMT crystals. However, for *x *> 0, the L1 and L2 luminescence lines begin to overlap and can no longer be resolved because of the increase of line broadening which accompanies the increase of *x*. The main emission peaks at the highest energy are believed to be due to the acceptor bound exciton (L1). The weak peak at slightly lower energies, 1.612 and 1.681 eV for *x *= 0.05 and 0.11 in Figure 1 has been attributed to the electron-to-neutral acceptor transitions (e, A^0^). The energy separation between L1 and (e, A^0^) increases from 46 to 66 meV upon increasing *x *from 0.05 to 0.11, which is larger than the separation of 41 meV for CdTe [10]. In addition, the peak at 1.728 eV for *x *= 0.11 is the LO-phonon replica of the L1 transition giving a LO phonon energy of 21 meV in agreement with the published value of 21.4 meV [11]. The L1 peaks (as well as L2 when they are resolved) are blue shifted with increasing Mn mole fraction *x*. According to Lautenschlager et al. [12], this blueshift is responsible to the decrease of lattice constant, which is not strongly affected by the admixture of Mn 3*d *states. As the incorporation of Mn atoms increases, the band-edge luminescence peak becomes broader and weaker.

**Figure 2:**
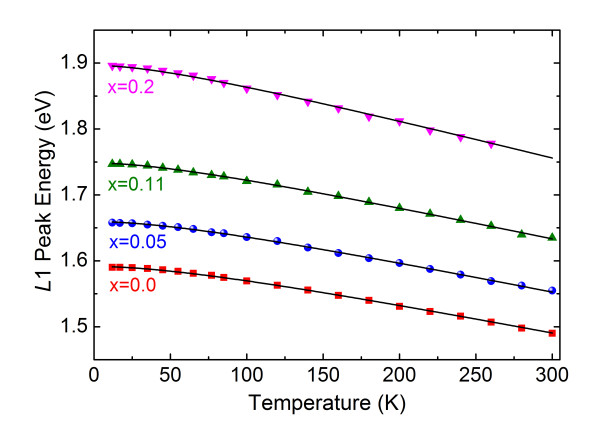
**Temperature dependence of the neutral acceptor-bound exciton peak L1 for a series of Mn concentrations**. The solid lines are the fits according to the Varshni relation (Equation 1).

Figure 2 shows the temperature dependences of the L1 peak positions for a series of alloys with different Mn concentrations. The observed band-edge luminescence energy shows a clear blueshift with decreasing *T*. The solid curves in the figures were drawn by fitting to the L1 emission peak positions the empirical equation [13]

(1) $$E_{0}\left(T\right)=E_{0}\left(0\right)-\alpha T^{2}∕\left(\beta +T\right),$$

where *E*_0_(0) is the transition energy at *T *= 0 K, and *α *and *β *are constants referred to as Varshni thermal coefficients. The parameter values of *α *and *β *obtained by fits to our *E*_0_(*T*) data with Equation 1 are listed in Table 1. The parameter *α *is seen to increase linearly with *x*. The change of the band-gap energy *E*_g _with temperature is usually attributed to the effect of electrons or phonons. The electron-hole pair is believed to soften the lattice vibration of the crystal when it is excited across a band gap [14]. This change in lattice vibration frequency can also occur when carriers are bound to vacancies or when impurities are excited. The increase in the temperature parameter *α *with increasing *x *in CMT indicates that the crystal is undergoing changes in the lattice vibration frequency as the Mn content increases in the alloy lattice. One can argue that the introduction of Mn into the CdTe lattice leads to the formation of vacancy (acceptor, donor) sites on which electrons or holes can be trapped. The presence of a trapped carrier reduces the interatomic force constant at or around the vacancy site, thus softening the normal-mode frequencies of the lattice and reducing the zero-point energy of the oscillators. An increase in the number of Mn atoms in the lattice seems to lead to an increase in the effective mass of the bound trap complex, and thus to an increase in mode softening [15]. This fact agrees with the observed increase in the parameter *α *with increasing *x *in Zn_1-*x*_Mn*_x_*Se [16]. The decrease of *β *seen in Table 1 is another feature characteristic of Mn-based DMS alloys [16]. It is well known that in a semiconductor, when the temperature is much below the Debye temperature, a *T*^2 ^dependence of the energy gap is observed, a behavior which led Varshni to propose a relation between the parameter *β *in Equation 1 and Debye temperature *θ*_D _[13]. In Mn-based DMSs the Debye temperature does not change with increasing Mn concentrations [17]. We therefore believe that there is an additional factor which increases the gap. Alexander et al. [18] analyzed this phenomenon as being due to the shift of the conduction band energy resulting from the exchange interaction between the magnetic ions and conduction electrons. We are thus inclined to conclude that the decrease in *β *with *x *is connected with a smooth shift of the conduction-band energy [19].

**Table 1:** Values of the parameters obtained by fitting the energy gap vs temperature to Equation 1

Mn	*E*_0_	*α*	*β*	-*dE*_g_/*dT *(77 ≤ *T *≤ 300)
*x*	(eV)	(10^-4 ^eV/k)	(K)	(10^-4 ^eV/k)
0.0	1.592	0.00046	110	3.94
0.05	1.660	0.00048	104	4.07
0.11	1.748	0.0005	91	4.36
0.2	1.896	0.00059	78	5.25

**Figure 3:**
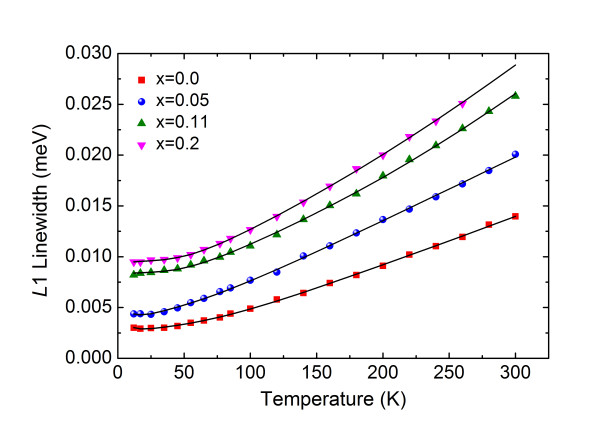
**Temperature dependence of the FWHM of L1 emission lines for a series of Mn concentrations**.

Figure 3 shows the temperature dependence of the full width at half maximum [FWHM] of the L1 emission line with Mn concentration *x*. With increasing Mn mole fraction *x*, the FWHM of the L1 emission line varies from approximately 3.02 meV for *x *= 0.0 to approximately 9.49 meV for *x *= 0.2. The FWHM of the L1 emission line also increases with increasing temperature, which can be accounted for by increasing the exciton-phonon interaction at higher temperatures. The temperature dependence of the emission linewidth caused by exciton-phonon interactions has the form [20]

(2) $$\Gamma \left(T\right)=\Gamma_{\text{inh}}+\gamma_{\text{LA}}T+\frac{\Gamma_{\text{LO}}}{\exp\left(\hbar \omega_{\text{LO}}∕k_{\text{B}}T\right)-1}+\Gamma_{\text{i}}\exp\left(E_{\text{i}}∕k_{\text{B}}T\right),$$

where *Γ*_inh _is the inhomogeneous broadening term, *γ*_LA _is a coefficient of exciton-acoustic phonon interaction, *Γ*_LO _is the exciton-LO phonon coupling constant, *ħω*_LO _is the LO phonon energy, *Γ*_i _is a proportionality factor which accounts for the concentration of impurity centers, and *E*_i _is the binding energy of impurity-bound excitons averaged over all possible locations of the impurities. The solid lines in Figure 3 are drawn by fitting Equation 2 to the experiment data. In the case of CdTe, the fitted values are *Γ*_inh _= 1.15 meV, *Γ*_LO _= 25.6 meV, *ħω*_LO _= 20.9 meV, and *γ*_LA _= 4.9 × 10^-6 ^meV. It is noted here that the contribution of the impurity scattering process to the linewidth broadening can be neglected. The value for *ħω*_LO _is in good agreement with the LO phonon energy of 21.3 meV in CdTe [6]. Listed in Table 2 are the obtained values of *Γ*_inh_, *Γ*_LO_, *ħω*_LO_, and *γ*_LA _for the Cd_1-*x*_Mn*_x_*Te crystals. We noted that the effect of *γ*_LA _is smaller than that of any other broadening term for each composition *x*, indicating that the effect of exciton-acoustic phonon interaction on the linewidth is negligible. It is noted that *Γ*_inh _and *Γ*_LO _increase with increasing *x*, while *ħω*_LO _is nearly constant. It is well known that *Γ*_LO _should increase with the polarity of the material and that the inhomogeneous broadening in *Γ*_inh _may be accounted for implicitly and partly by the presence of various site symmetries of the Mn^2+ ^ion in the lattice, the existence of stacking faults [21], and the presence of impurity complexes involving lattice vacancies [22].

**Table 2:** Values of *Γ*_inh_, *Γ*_LO_, *ħω*_LO_, and *γ*_LA _and the behaviors of activation energies

Mn	*Γ*_inh_	*Γ*_LO_	*ħω*_LO_	*γ*_LA_	*E*_1_	*E*_2_
*x*	(meV)	(meV)	(meV)	(10^-6 ^meV)	(meV)	(meV)
0.0	1.15	25.6	20.98	4.9	5.9	17.18
0.05	1.65	26.14	21.0	1.0	5.38	16.67
0.11	2.0	27.06	21.01	1.8	4.89	15.81
0.2	3.53	28.44	21.03	2.3	4.22	14.39

**Figure 4:**
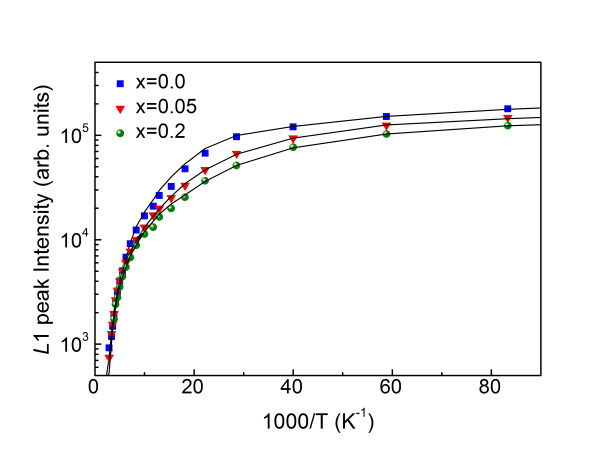
**The emission intensity of the neutral acceptor-bound exciton line L1**. The emission intensity is a function of reciprocal temperature for a series of Mn concentrations *x*.

Parameters on *풢*_inh_, *풢*_LO_, *ħω*_LO_, and *γ*_LA _as a function of Mn concentration *x *were obtained by fitting Equation 2 to the data shown in Figure 3. Also listed are the obtained values of *E*_1 _and *E*_2_, obtained by fitting Equation 3 to the data shown in Figure 4.

Figure 4 shows the L1 luminescence intensity of CMT crystals as a function of reciprocal temperature in the range from 12 K to 300 K. It is recognized that a host of different processes contribute to reduction of the PL intensity as temperature increases. As observed in other semiconductors [23], the temperature dependence of the luminescence intensity shows a two-step quenching process and can be expressed in the form

(3) I(T)=I01+C1 exp(-E1∕kT)+C2 exp(-E2∕kT),

where *E*_1 _and *E*_2 _are the high- and low-temperature activation energies, respectively. For CdTe, the values of the parameters obtained from the fit to Equation 3 are *E*_1 _= 5.9 meV and *E*_2 _= 14.3 meV. These values are in fairly good agreement with the reported exciton binding energies for the L1 line [24]. Listed in Table 2 are the behaviors of activation energies as a function of Mn mole fraction *x*. The activation energy is seen to decrease with increasing Mn concentration, similar to the results reported by Su et al. [25]. This behavior has been interpreted in terms of the four-center model suggested by Fonger [26] and observed in many ternary alloys [27,28].

## Conclusion

The PL characteristics of the diluted magnetic semiconductor alloy CMT crystals were systematically studied as a function of temperature and Mn concentrations. Bulk crystals were grown by the vertical Bridgman method for Mn concentrations from *x *= 0.0 to 0.2. Our studies involved near-band-edge excitonic PL emission of neutral acceptor-bound excitons with emphasis on the dependence of the PL spectrum on temperature and alloy composition. With increasing *x*, the Varshni parameter *α *increased while *β *decreased. From the temperature dependence of the FWHM of the L1 emission line, the broadening factors were determined by fitting Equation 2 to the data. From the temperature dependence of L1 we were able to determine the activation energies for thermal quenching of the corresponding bound excitons. Our results showed that the values of activation energies decrease with increasing Mn concentration *x*.

## Competing interests

The authors declare that they have no competing interests.

## Authors' contributions

YH carried out the CdMnTe crystal growth and wrote this manuscript. YU participated in the design of this research and performed the data analysis. HP participated in the measurement of photoluminescence and analysis. All authors read and approved the final manuscript.

## References

[B1] Furdyna JK Diluted magnetic semiconductors J Appl Phys 1988 64 R29 10.1063/1.341700

[B2] Kavokin KV Merkulov IA Yakovlev DR Ossau W Landwehr G Exciton localization in semimagnetic semiconductors probed by magnetic polarons Phys Rev B 1999 60 16499 16505 10.1103/PhysRevB.60.16499

[B3] Mycielski A Kowalczyk L Galazka RR Sobolewski R Wang D Burger A Sowinska M Groza M Siffert P Szadkowski A Witkowska B Kaliszek W Applications of II-VI semimagnetic semiconductors J Alloys Compd 2006 423 163 168 10.1016/j.jallcom.2005.12.116

[B4] Kim KH Camarda GS Bolotnikov AE James RB Hong J Kim S Improved carrier-transport properties of passivated CdMnTe crystals J Appl Phys 2009 105 093705 10.1063/1.3121502

[B5] Pavesi L Guzzi M Photoluminescence of Al*_x_*Ga_1-*x*_As alloys J Appl Phys 1994 75 4779 4842 10.1063/1.355769

[B6] Halliday DP Potter MDG Mullins JT Brinkman AW Photoluminescence study of a bulk vapour grown CdTe crystal J Crystal Growth 2000 220 30 38 10.1016/S0022-0248(00)00755-7

[B7] Palosz W Grasza K Boyd PR Cui Y Wright G Roy UN Burger A Photoluminescence of CdTe crystals grown by physical-vapor transport J Electron Mater 2003 32 747 751 10.1007/s11664-003-0064-8

[B8] Taguchi T Onodera C Shallow acceptor bound-excitons in CdTe Epitaxial layers on (100) GaAs Mat Sci Forum 1990 65 235 240

[B9] Francou JM Saminadayar K Pautrat JL Shallow donors in CdTe Phys Rev B 1990 41 12035 12046 10.1103/PhysRevB.41.12035 9993655

[B10] Oda O Compound semiconductor bulk materials and characterizations 2007 World scientific Publishing. Co. Pte. Ltd 422 424

[B11] Becla P Kaiser D Giles NC Lansari Y Schetzina JF Electrical and optical properties of P- and As-doped Cd_1-*x*_Mn*_x_*Te J Appl Phys 1987 62 1352 1362 10.1063/1.339638

[B12] Lautenschlager P Logothetidis S Viña L Cardona M Ellipsometric studies of the dielectric function of Cd_1-*x*_Mn*_x_*Te alloys Phys Rev B 1985 32 3811 3818 10.1103/PhysRevB.32.3811 9937531

[B13] Varshni YP Temperature dependence of the energy gap in semiconductors Physica 1967 34 149 154 10.1016/0031-8914(67)90062-6

[B14] Heine V Van Vechten JA Effect of electron-hole pairs on phonon frequencies in Si related to temperature dependence of band gaps Phys Rev B 1976 13 1622 1626 10.1103/PhysRevB.13.1622

[B15] Bottka N Stankiewicz J Giriat W Electroreflectance studies in Cd_1-*x*_Mn*_x_*Te solid solutions J Appl Phys 1981 52 4189 4193 10.1063/1.329233

[B16] Bylsma RB Becker WM Kossut J Debska U Dependence of energy gap on *x *and *T *in Zn_1-x_Mn_x_Se: the role of exchange interaction Phys Rev B 1986 33 8207 8215 10.1103/PhysRevB.33.8207 9938213

[B17] Galazka RR Nagata S Keesom PH Paramagnetic-spin-glass-antiferromagnetic phase transitions in Cd_1-*x*_Mn*_x_*Te from specific heat and magnetic susceptibility measurements Phys Rev B 1980 22 3344 3355 10.1103/PhysRevB.22.3344

[B18] Alexander S Helman JS Balberg I Critical behavior of the electrical resistivity in magnetic systems Phys Rev B 1976 13 304 315 10.1103/PhysRevB.13.304

[B19] Diouri J Lascaray JP El Amrani M Effect of the magnetic order on the optical-absorption edge in Cd_1-x_Mn_x_Te Phys Rev B 1985 31 7995 7999 10.1103/PhysRevB.31.7995 9935746

[B20] Rudin S Reinecke TL Segall B Temperature-dependent exciton linewidths in semiconductors Phys Rev B 1990 42 11218 11231 10.1103/PhysRevB.42.11218 9995407

[B21] Lanver U Lehmann G Luminescence spectra of Mn(II) in different symmetries J Lumin 1978 17 225 235 10.1016/0022-2313(78)90088-1

[B22] Sibley WA Koumvakalis N Perturbed Mn^2+ ^transitions in irradiated RbMgF_3_:Mn Phys Rev B 1976 14 35 40 10.1103/PhysRevB.14.35

[B23] Bimberg D Sondergeld M Grobe E Thermal dissociation of excitons bounds to neutral acceptors in high-purity GaAs Phys Rev B 1971 4 3451 3455 10.1103/PhysRevB.4.3451

[B24] Cohen E Street RA Muranevich A Bound excitons and resonant Raman scattering in Cd*_x_*Zn_1-*x*_Te(0.9*≤x≤*1) Phys Rev B 1983 28 7115 7124 10.1103/PhysRevB.28.7115

[B25] Su JS Wang JC Chen YF Shen JL Chou WC Dielectric studies of Zn_1-*x*_Mn*_x_*Se epilayers J Appl Phys 1999 86 1630 1633 10.1063/1.370938

[B26] Fonger WH Nearest-neighbor splitting of the luminescence levels of ZnS*_x_*Se_1-*x*_ Phys Rev 1965 137 A1038 A1048 10.1103/PhysRev.137.A1038

[B27] Block D Cox RT Optically detected magnetic resonance of donor-a center pairs in ZnS_x_Se_1-x _(x = 0.995) J Lumin 1981 24-25 167 171

[B28] Zunger A Composition-dependence of deep impurity levels in alloys Phys Rev Lett 1985 54 849 10.1103/PhysRevLett.54.849 10031634

